# A Study on the Sufficient Conditional and the Necessary Conditional With Chinese and French Participants

**DOI:** 10.3389/fpsyg.2022.787588

**Published:** 2022-02-24

**Authors:** Jing Shao, Dilane Tikiri Banda, Jean Baratgin

**Affiliations:** ^1^Laboratory Cognition Humaine et Artificielle, Université Paris 8, Paris, France; ^2^Université de Haute-Alsace, Mulhouse, France; ^3^Probability, Assessment, Reasoning and Inferences Studies (P-A-R-I-S) Association, Paris, France

**Keywords:** Sapir-Whorf hypothesis, universalist hypothesis, cross-cultural comparison, sufficient conditional, necessary conditional, deduction under uncertainty, de Finetti’s coherence

## Abstract

According to the weak version of linguistic relativity, also called the Sapir-Whorf hypothesis, the features of an individual’s native language influence his worldview and perception. We decided to test this hypothesis on the sufficient conditional and the necessary conditional, expressed differently in Chinese and French. In Chinese, connectors for both conditionals exist and are used in everyday life, while there is only a connector for the sufficient conditional in French. A first hypothesis follows from linguistic relativity: for the necessary conditional, better logic performance is expected in Chinese participants rather than French participants. As a second hypothesis, for all participants, we expect performance on the sufficient conditional to be better than on the necessary conditional. Indeed, despite the isomorphism of the two conditionals, they differ in how information is processed for reasoning. We decided to study reasoning under uncertainty as it reflects reality more accurately. To do so, we analyzed the coherence of participants using de Finetti’s theory for deduction under uncertainty. The results of our study show no significant difference in performance between Chinese and French participants, neither on the sufficient conditional nor on the necessary conditional. Thus, our first hypothesis derived from the weak version of linguistic relativity is not confirmed. In contrast, our results confirm the second hypothesis in two out of three inference schemas.

## Introduction

### The Sapir-Whorf Hypothesis

For decades, linguistic relativity theory, also known as the Sapir-Whorf hypothesis, has been omnipresent in studying the relationship between thought and language. Linguistic relativity theory, defended by Sapir (1921) and more radically by Whorf (1956), proposes that language influences the way people perceive and think about the world. This hypothesis focuses on the differences in both vocabulary and grammar between languages. It suggests that people’s language vocabulary and grammatical structure strongly influence how they conceptualize the world. Whorf considers that human language has an additional role in shaping thought besides its function as a communication tool. Two versions of the principle of linguistic relativity can be distinguished: the weak version and the strong version (Carnes, 1970; Brown, 1976). According to the strong version, the characteristics of our native language *determine* our worldview and way of perceiving; as for the weak version, the former *influences* the latter. The strong version refers to linguistic determinism. Whorf himself does not make such a distinction. As Yao (2002) has pointed out, Whorf sometimes favored the weak version, sometimes the strong one. Compared to the strong version, which is very radical and that most researchers do not adhere to, the weak version seems much more realistic.

Whorf (1956) claims that grammatically based systems that differ across languages exercise an unconscious control over reasoning; that is, the grammar of one’s native language might affect one’s reasoning. Precisely, one’s reasoning competence would be constrained by the presence or absence of grammatical structures in one’s mother tongue. Counterfactual reasoning is an important topic in this area of research. Bloom (1981, 1984) proposed that Chinese speakers lacked a specific counterfactual construction without a distinct counterfactual marker (the subjunctive). For Bloom, this leads to a reduced ability to engage in counterfactual reasoning for Chinese speakers, compared to English speakers, who have a subjunctive structure. His results confirm the weak version of the Sapir-Whorf hypothesis. In contrast, Au (1983, 1984) and Liu (1985) did not find any particular difficulty of Chinese speakers with counterfactual reasoning compared to English speakers. Takano (1989) did not find any difference between Japanese speakers, who similarly lack a counterfactual marker, and English speakers. Their results invalidate the Sapir-Whorf hypothesis. However, more recently, the result of Yeh and Gentner (2005) has partly validated the weak version of the Sapir-Whorf hypothesis. In their experiment, when the participants had sufficient knowledge to interpret a counterfactually presented portion of a story, there was no difference between Chinese and English speakers. When they did not, the results showed an advantage for English speakers over Chinese speakers. As for the interference between thought and language, Hunt and Agnoli (1991) have argued that the locus of the interference between thought and language would not lie at the conceptual level but instead at the information processing level.

### Sufficient Conditional and Necessary Conditional

In the same manner, as with counterfactual reasoning, we would like to test the validity of the weak version of the Sapir-Whorf hypothesis. We compared Chinese and French speakers regarding the sufficient conditional and the necessary conditional in our experiment. The sufficient conditional refers to the reasoning “if A, then C,” which means that, given the antecedent A, the consequent C occurs. As for the necessary conditional, it refers to “only if A, then C,” which implies that the antecedent A is necessary for the consequent C to happen. A’s presence is required to make C happen but might not be enough, unlike the sufficient conditional. The two conditionals are not expressed identically in Chinese and French. On the one hand, in Chinese, both connectors for the sufficient conditional and the necessary conditional are present in daily life; on the other hand, only a connector for the sufficient conditional exists in French. According to the Sapir-Whorf hypothesis, Chinese participants should perform better than French participants on the necessary conditional, given the presence of the corresponding connector in their mother tongue. Also, there should be no significant difference between Chinese and French participants concerning the sufficient conditional, given that the connector for the sufficient conditional is present and widely used in both languages. In addition, we state a second hypothesis that there should be better performance in the sufficient conditional than in the necessary conditional. In the necessary conditional “only if A, then C,” the antecedent A is necessary for the consequent C. This means that, without the presence of A, there is no C. So, “Only if A, C” is equivalent to “If not-A, then not-C” (Johnson-Laird and Byrne, 1991; Gomes, 2009; Wang and Gao, 2010), which has the same structure as the sufficient conditional “If P, then Q” (P being not-A, Q being not-C). Thus, the two conditionals can be interpreted as isomorphic. Nevertheless, the information processing most likely differs between the two conditionals, as reasoning in the necessary conditional *a priori* implies the process of transformation to the sufficient conditional in our experiment, in addition to reasoning in the sufficient conditional. We make such a claim due to the nature of the necessary conditional, which does not guarantee any event; it does not lead to another result in general, which makes the reasoning more difficult.

In Chinese communication, the sufficient conditional with the connector “*rúguǒ* A, *nàme* C” translates into “If A, then C” and the necessary conditional with the connector “*zhǐyǒu* A, *cáihuì* C” translates into “Only if A, C.” Most studies on conditional reasoning have focused on the sufficient conditional “if A, then C.” There are few studies on the necessary conditional “A, only if C,” logically equivalent to the sufficient conditional “if A, then C” (Evans, 1977; Evans and Beck, 1981; Grosset and Barrouillet, 2003). Those studies examined whether both conditionals were interpreted similarly by the participants. Despite logical equivalence, the results showed that those two forms seemed to be interpreted differently by the participants (Evans, 1977; McCawley, 1981). “If A, then C” is not always interpreted as “A, only if C”: it is sometimes interpreted as “A, only if C,” and sometimes as “C, only if A.” Evans (1977); Evans and Newstead (1977), and Evans and Beck (1981) considered that the “only if” syntax involves both a temporal and a necessity relation. Thompson and Mann (1995) consider that pragmatic contexts, such as in the interpretation of necessity and temporal relations, might play a more indirect role. From another perspective, the study of Wang and Gao (2010) consisted in comparing the performance of Chinese participants with the traditional inference schemas: Modus Ponens (MP), Modus Tollens (MT), Denying the Antecedent (DA), and Affirmation of the Consequent (AC), with the sufficient conditional “If A, then C” and the necessary conditional “Only if C, A” logically equivalent. By way of a reminder, MP denotes the reasoning from a premise “if A, then C,” knowing the event A occurs. MT implies reasoning from the same premise, considering the event C does not occur. Likewise, DA refers to a situation where A does not occur, and AC to a condition in which C occurs. Their study showed a significant effect of the representation of semantic relations on conditional inferences. For example, for MP, the rate of correct response (73.8%) with “If A, then C” was much higher than the rate (47.7%) with “Only if C, A,” despite the logical equivalence of both inferences. To interpret this result, the authors explained that, in the “If A, then C” form, the sufficiency of *A* for *C is* explicit, whereas in the necessary conditional “Only if C, A,” it is implicit. The participants performed better on conditional inferences corresponding to explicit semantic relations than those corresponding to implicit semantic relations. It should be noted that all the studies so far on the necessary conditional, including the studies cited above, investigated reasoning under certainty, which means reasoning from certain assumption. As for us, we decided to study the reasoning on the necessary conditional under uncertainty, implying the possibility that the assumptions might not certainly happen, as it reflects reality more accurately.

As one should note, the necessary conditional statement “Only if A, C” in Chinese is different from the statement “A, only if C.” Firstly, there is a difference of directionality: “Only if A, C” in Chinese starts from the antecedent, and consists in deducing the consequent, from the antecedent, whereas “A, only if C” starts from the consequent, and consists in inferring the antecedent. Numerous studies in the context of certainty have underlined a directionality effect, which means people perform better while making inferences that correspond to the direction of the conditional (Evans, 1977, 1993; Evans and Beck, 1981; Johnson-Laird and Byrne, 1991; Ormerod et al., 1993; Rips, 1994; Grosset and Barrouillet, 2003; Byrne and Johnson-Laird, 2009). Secondly, the necessary conditional “Only if A, C” in Chinese is used as such in daily life. Thus, we deem it natural and relevant to study the Chinese necessary conditional as it appears in Chinese: “Only if A, C,” instead of “Only if C, A.” The necessary conditional “Only if A, C” implies the sufficient conditional “If not-A, then not-C.” Our study on the sufficient conditional and the necessary conditional offers us twice as many situations to study the reasoning as the classical study of the sole sufficient conditional.

### Reasoning Under Uncertainty: A New Paradigm

In the study of inferences, we classically set the premise as certain, but this rarely occurs in everyday life. Hence, we decided to use a framework to consider uncertainty in human reasoning. We have opted for the new paradigm approach of reasoning (Oaksford and Chater, 2007, 2009; Over, 2009; Evans, 2012; Elqayam and Evans, 2013; Elqayam and Over, 2013; Evans and Over, 2013; Mandel, 2014), which highlights the importance of uncertainty in human deductive reasoning. In this approach, the reference model is no longer binary logic but the Bayesian model. More specifically, in our study, we adopt the subjective Bayesian theory of De Finetti (1964), which has many theoretical, methodological, and prescriptive advantages (Baratgin and Politzer, 2016; Over and Baratgin, 2016; Politzer and Baratgin, 2016; Baratgin et al., 2017; Oaksford and Chater, 2020; Politzer et al., 2020a,b; Baratgin, 2021; Lassiter and Baratgin, 2021).

Theoretically, the Finettian approach is based on the Bayesian subjective concept of coherence, which states that the degrees of belief must respect the axioms of probability (Baratgin, 2002; Baratgin and Politzer, 2006). The theory of De Finetti (1980) distinguishes two levels of experimental analysis, corresponding to two levels of knowledge of an event. The elementary level concerns the belief in the realization of some event *C* conditioned on the state of knowledge of some individual *A* (noted *C|A*). *C|A* is a tri-event having three values of truth: true when *A* and *C* are true, false when A is true and C is false, and uncertain when A is uncertain or false. Recent studies (Politzer et al., 2010; Baratgin et al., 2013, 2014, 2018; Nakamura et al., 2018) have shown that most participants interpret the conditional of natural language in the same way as indicated in the theory of De Finetti (1995). The epistemic meta-level relates to the degrees of belief in the event. Many studies have shown the strong acceptance of participants to the principal property of this level, that the probability of the indicative conditional “if A, then C” is equal to the conditional probability *P(C|A)* (Evans and Over, 2004; Oaksford and Chater, 2007, 2009; Pfeifer and Kleiter, 2010; Politzer et al., 2010; Manktelow, 2012). More recently, there have been advances in the study of human coherence in deduction under uncertainty (Pfeifer and Kleiter, 2011; Pfeifer, 2014; Singmann et al., 2014; Cruz et al., 2015; Evans et al., 2015; Politzer and Baratgin, 2016). De Finetti (1964, 1974) provides an effective method to appraise the coherence of a probability evaluation, using coherence intervals determined by the probability of the premises (Suppes, 1966; Hailperin, 1996, 2010; Coletti and Scozzafava, 2002; Gilio and Over, 2012; Baratgin and Politzer, 2016; Politzer, 2016). If the coherence interval of the conclusion is [0, 1], the inference schema is called “probabilistically uninformative”; if the coherence interval of the conclusion is a restrained interval [l, u], it is called “probabilistically informative” (Pfeifer and Kleiter, 2006). Pfeifer and Kleiter (2007) used this methodology to study inference schemas MP and DA. In their experiment on MP, the inference schema was probabilistic because they used statements such as “exactly 80% of the red cars on this parking lot are two-door cars, exactly 90% of the cars on this parking lot are two-door cars,” and the question “Imagine all the cars that are on this parking lot. How many of these cars are two-door cars?” 63% of the participants gave coherent intervals for MP, only 41% for DA. The results for MP are in line with the pioneering study by George (1997) (see also Singmann et al., 2014; Evans et al., 2015, for similar results).

In the context of uncertainty, we decided to study three inference schemas, among which the two main classical ones: the probabilistic inference schema for MP, called PMP, covering from DA to MP; the probabilistic inference schema for AC, called PAC, covering from MT to AC. Besides PMP and PAC inference schemas, we also studied a third inference schema, IF-introduction: “A, C, therefore, if A then C” in probabilistic form, called PIF. Table 1 shows probabilistic inference schemas in the sufficient conditional “If A, then C” and the necessary conditional “Only if A, C.”

**TABLE 1:** Probabilistic inference schemas in the sufficient conditional “If A, then C” and the necessary conditional “Only if A, C”.

Probabilistic inference schemas	Sufficient conditional “If A, then C”	Necessary conditional “Only if A, C”
PMP	*P*(If A, then C), *P*(A) ⇒*P*(C)	*P*(Only if A, C), *P*(A) ⇒*P*(C)
PAC	*P*(If A, then C), *P*(C) ⇒*P*(A)	*P*(Only if A, C), *P*(C) ⇒*P*(A)
PIF	*P*(A), *P*(C) ⇒*P*(If A, then C)	*P*(A), *P*(C) ⇒*P*(Only if A, C)

We thus have a kind of ‘‘trilogy,’’ in which the premises are taken in pairs out of a set of three sentences (A, C, and ‘‘if A, C’’).^1^

In this study, we analyze the performances in terms of coherence, for Chinese and French participants, in these three inference schemas with two conditional forms: the sufficient conditional “If A, then C” and the necessary conditional “Only if A, C.” The coherence interval for the conclusions of MP and AC can be obtained by calculation (Suppes, 1966; Hailperin, 1996, 2010; Coletti and Scozzafava, 2002; Gilio, 2002; Wagner, 2004; Sobel, 2009) or by an analogical representation method (Politzer, 2016). We present the coherence intervals for the three inference schemas in each, the sufficient conditional and the necessary conditional.

In the sufficient conditional, the probabilistic inference schema for MP (PMP), which can be obtained from the probability of the conditional and the probability of the antecedent, is written:


$$\begin{array}{c} P\left(ifA,thenC\right)=i\\ \frac{P\left(A\right)=a}{a\times i\leq P\left(C\right)\leq a\times i+1-a} \end{array}$$

When *i = 1, a = 0*, we are in the particular situation that corresponds to classical DA, and when *i = 1, a = 1*, we are in the particular situation of classical MP.

The probabilistic inference schema AC (PAC), obtained from the probability of the conditional and the probability of the consequent, is written:


$$\begin{array}{c} P\left(ifA,thenC\right)=i\\ \frac{P\left(C\right)=c}{0\leq P\left(A\right)\leq min\left\{\frac{c}{i},\frac{1-c}{1-i}\right\}} \end{array}$$

when *i = 1, c = 0*, we are in the particular situation that corresponds to classical MT, and when *i = 1, c = 1*, we are in the particular situation of classical AC.

The probabilistic inference schema IF-introduction (PIF), which can be obtained from the probability of the antecedent and the probability of the consequent, is written:


$$\begin{array}{c} P\left(A\right)=a\\ \frac{P\left(C\right)=c}{max\left\{0,\frac{c-1+a}{a}\right\}\leq P\left(ifA,thenC\right)\leq min\left\{\frac{c}{a},1\right\}} \end{array}$$

We examined the case of the necessary conditional “Only if A, C.” “Only if A, C” corresponds to “If not-A, then not-C” in the sufficient conditional. The probability of the conditional “Only if A, C” is that of the sufficient conditional “If not-A, then not-C,” the probability of the antecedent is *P*(A), and the probability of consequence is *P*(C).

Thus, the inference schema MP in the necessary conditional « “*Only if A, C*,” *A* » corresponds to DA « “If not-A, then not-C,” A » in the sufficient conditional. The probabilistic inference schema for MP (PMP) in necessary conditional is the probabilistic inference schema for DA (PDA) in the sufficient conditional, which is written as follows:


$$\begin{array}{c} P\left(if\;not-A,then\;not-C\right)=i\\ \frac{P\left(A\right)=a}{\left(1-a\right)\left(1-i\right)\leq P\left(C\right)\leq 1-\left(1-a\right)\times i} \end{array}$$

when *i = 1, a = 0*, we are in the particular situation of DA in the necessary conditional that corresponds to classical MP in the sufficient conditional. When *i = 1, a = 1*, we are in the particular situation of MP in the necessary conditional that corresponds to classical DA in the sufficient conditional.

In the same way, the inference schema PAC in the necessary conditional corresponds to PMT in the sufficient conditional. Thus, the inference schema AC in the necessary conditional « “Only if A, C,” C » corresponds to MT « “If not-A, then not-C,” C » in the sufficient conditional. The probabilistic inference schema for AC (PAC) in necessary conditional is the probabilistic inference schema for MT (PMT) in the sufficient conditional, which is written as follows:


$$\begin{array}{c} P\left(if\;not-A,then\;not-C\right)=i\\ \frac{P\left(C\right)=c}{max\left\{\frac{c-i}{1-i},\frac{i-c}{i}\right\}\leq P\left(A\right)\leq 1} \end{array}$$

when *i = 1, c = 0*, we are in the particular situation of MT in the necessary conditional that corresponds to classical AC in the sufficient conditional. When *i = 1, c = 1*, we are in the particular situation of AC in the necessary conditional that corresponds to classical MT in the sufficient conditional.

Because the probability of the necessary conditional “only if A, C” corresponds to the probability of the sufficient conditional “If not-A, then not-C,” which is *P* [(1-c)/(1-a)], the probabilistic inference schema IF-introduction (PIF) in necessary conditional is written as follows:


$$\begin{array}{c} P\left(A\right)=a\\ \frac{P\left(C\right)=c}{max\left\{0,\frac{1-a-c}{1-a}\right\}\leq P\left(if\;not-A,then\;not-C\right)}\\ \leq min\left\{\frac{1-c}{1-a},1\right\} \end{array}$$

## Experiment

On the one hand, our goal was to test the Sapir-Whorf hypothesis by comparing the percentage of coherence of Chinese and French participants in both the sufficient conditional and the necessary conditional. On the other hand, we expected a better performance in the sufficient conditional than in the necessary conditional. Despite isomorphism of the sufficient conditional and the necessary conditional, the two conditionals might involve different information processing, resulting in better performances for the sufficient conditional. Indeed, it is likely that reasoning in the necessary conditional would imply *a priori* the transformation process to the sufficient conditional and reasoning in the sufficient conditional.

In this study, we took the methodology used in Politzer and Baratgin (2016). The uncertainty of the premises as the choices of answers provided for the participants is formulated in a qualitative form, in contrast with a numerical form (a value between 0 and 1, or in the form of a percentage) as used in most previous studies on PMP and PAC (Pfeifer and Kleiter, 2009, 2010, 2011; Pfeifer, 2014; Singmann et al., 2014; Cruz et al., 2015; Evans et al., 2015; Nickerson et al., 2019). This methodology is consistent with the subjective conception of de Finetti’s theory. Moreover, we believe that in everyday life, people do not reason by assigning a quantitative probability to an event or a conditional, but a qualitative probability such as high, medium, and low as de Finetti suggested himself (De Finetti, 1964; Baratgin and Politzer, 2006, 2007).

### Methods

#### Material

In our pilot experiment carried out on the sufficient conditional in France, the participants had to deal with two probabilistic inference schemas: PMP and PAC. The probability of the major premise as that of the minor premise varied from 0% to 100%, passing through low, medium, and high. We found out that when the two premises are uncertain, with verbal probability (probability of the first premise: high/medium/low, and probability of the minor premise: high/medium/low), most participants were confused, they had difficulty choosing their responses, the answers were primarily given randomly. Therefore, we decided not to combine two uncertain probabilities in our experiment. Indeed, we had the apprehension that the participants in the whole experiment would randomly select their answers instead of reasoning.

In our questionnaire, each item had two premises, a first premise and a second premise, for which we varied the levels of uncertainty: 100%, high, medium, low, 0%. When the first premise’s value was 100% or 0%, the second premise’s value was 100%, high, medium, low, or 0%. When the first premise’s value was high, medium, or low, the second premise’s value was 100% or 0%.

As in the experiments of Politzer and Baratgin (2016), it was followed by a multiple-choice response format.

When the first premise and the second premise are both certain (*0%* or *100%)*, the response options are:

*- exactly 0%* and *above 0%*, when the second premise is *0%*;
*- exactly 100%* and *below 100%*, when the second premise is *100%.*

When one of the premises is *high*, *medium*, or *low*, there are three response options, depending on the degree of uncertainty of the uncertain premise:

*- above* [*the level of the uncertain premise*];
*- just* [*the level of the uncertain premise*];
*- below* [*the level of the uncertain premise*].

For example, if the first premise is 100% and the second premise is high, the response options are *above high*, *just high*, and *below high*. In this situation, there were seven possible responses from participants: *above; just; below* (only one primitive option at a time); *above and just; below and just; above and below* (two primitive options); and *above, just*, *and below* (all three primitive options). Table 2 summarizes the design of the items.

**TABLE 2:** Response format according to the level of uncertainty of the premises.

		First premise				
		100%	High	Medium	Low	0%
**Second premise**	100%	- Exactly 100% - Below 100%	- Above high - Just high - Below high	- Above medium - Just medium - Below medium	- Above low - Just low - Below low	- Exactly 100% - Below 100%
	High	- Above high - Just high - Below high				- Above high - Just high - Below high
	Medium	- Above medium - Just medium - Below medium				- Above medium - Just medium - Below medium
	Low	- Above low - Just low - Below low				- Above low - Just low - Below low
	0%	- Exactly 0% - Above 0%	- Above high - Just high - Below high	- Above medium - Just medium - Below medium	- Above low - Just low - Below low	- Exactly 0% - Above 0%

The point of this multiple-choice format is that it makes the ideas of De Finetti (1980) explicit by differentiating between certainty, where one is certain that an event is true or false, whether or not it is verified, and subjective uncertain judgments. Thus, 0% and 100% are used to indicate certainty with extreme objectivity to avoid confusion and qualitative probability to express uncertainty. Therefore, this response format we used is not an ordinary mixture of numerical and verbal responses.

Each participant had to deal with one of eight different questionnaires: 4 with the sufficient conditional and 4 with the necessary conditional. For each questionnaire, questions were presented in 2 counterbalanced orders. Every questionnaire included 12 questions. In each questionnaire, the participants had to treat the 3 probabilistic inference schemas: PMP, PAC, and PIF. The participants were asked to select all the options that seemed correct. Here is an example of a question for PMP in the sufficient conditional:

*Knowing that the chances that “If Sophie is in the living room, then Mary is in the kitchen” are 100%*,

*knowing that the chances are low that “Sophie is in the living room.”*

*In your opinion, the chances that “Mary is in the kitchen” are:*

□ *above low*

□ *just low*

□ *below low*

The ordinal judgment “low” is considered as equivalent to the numerical probability 1/4 for us, “medium” is considered as similar as 1/2, and “high” is considered as 3/4. In this example, the first premise P(C/A) is 1 and the second premise *P*(A) is considered equivalent to 1/4. When we use the PMP formula mentioned in the previous part, *a* × *i* ≤ *P*(*C*) ≤ *a* × *i* + 1−*a*, we find the interval [1/4, 1]. Therefore, the coherent responses are “just low” and “above low.” We may also translate “low” into 0.20, “medium” into 0.50, and “high” into 0.80. We consider “low” as a probability of less than 50%, “medium” as a probability of 50%, and “high” as a probability of more than 50%.

It should be noted that in the questions, there was no causality between the antecedent and the consequent. Furthermore, to study only the logical aspect of reasoning, we paid attention to the choice of the first names, the gender, and the actions to prevent stereotypes from intervening.^2^

#### Participants

The Chinese participants were 295 students in the first and second grades of “media management” at Zhejiang University of Media and Communications in China. They were all native speakers of Chinese. The age of the participants extended from 18 to 23, with a mean age of 19.3. The French participants were 242 students, mainly from Universities Paris 1, Paris 5, Paris 8, and the others being students or former students of other universities in Paris. They were all native speakers of French. The age of the participants extended from 18 to 27, with a mean age of 20.3. Education levels ranged from high school diplomas to master’s diplomas. The participants voluntarily took part in the experiment and gave their consent to participate in it. None of them were trained in logic. The participants were not screened for knowledge of other languages than the one classified as their mother tongue, and it was assumed each participant would only have one mother tongue. We used the criterion of the mother tongue because we wanted to test the Sapir-Whorf hypothesis, which focuses on an individual’s native language. The duration of the test was 15 min.

### Results

#### Comparison of Coherence Between the Chinese and the French Participants

If a participant’s response is within the coherence interval, it is considered coherent.

##### Sufficient Conditional

**FIGURE 1:**
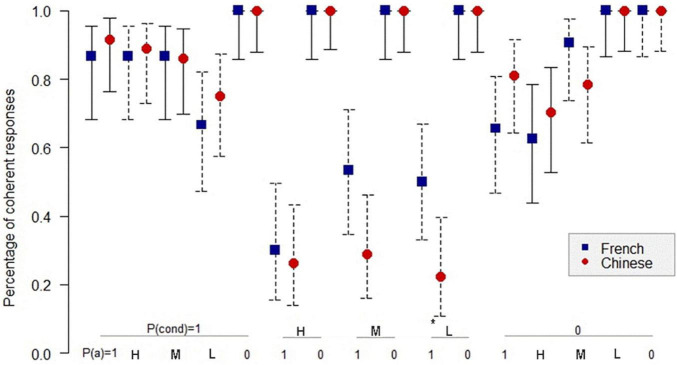
Percentage of coherent responses for the Chinese and the French participants in PMP in the sufficient conditional. *: *p* < 0.05. On the abscissa, the probabilities of the conditional and the probabilities of the antecedent below; on the ordinate: the percentage of coherent response for the Chinese and the French participants. Uninformative cases are those where the percentage of coherent responses is 1.0 for the French and the Chinese participants. MP: *P*(cond) = 1, *P*(a) = 1; DA: *P*(cond) = 1, *P*(a) = 0. The bars indicate the 95% confidence intervals for proportions.

**FIGURE 2:**
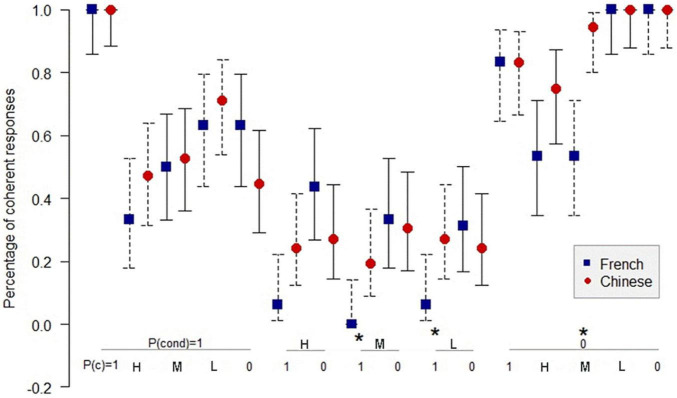
Percentage of coherent responses for the Chinese and the French participants in PAC in the sufficient conditional. *: *p* < 0.05. On the abscissa, the probabilities of the conditional and the probabilities of the consequent below; on the ordinate, the percentage of coherent response for the Chinese and the French participants. Uninformative cases are those where the percentage of coherent responses is 1.0 for the French and the Chinese participants. MT: *P*(cond) = 1, *P*(c) = 0; AC: *P*(cond) = 1, *P*(c) = 1. The bars indicate the 95% confidence intervals for proportions.

**FIGURE 3:**
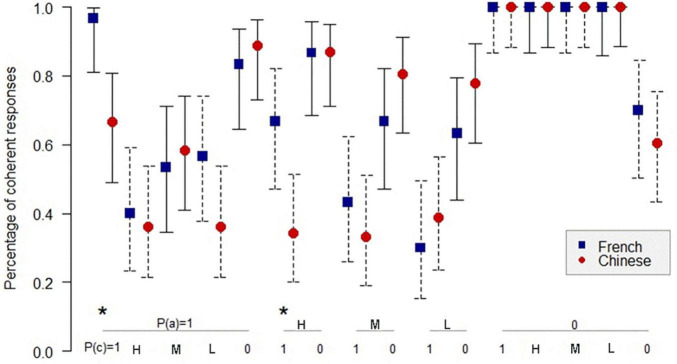
Percentage of coherent responses for the Chinese and the French participants in PIF in the sufficient conditional. *: *p* < 0.05. On the abscissa: the probabilities of the antecedent and the probabilities of the consequent below; on the ordinate: the percentage of coherent response for the Chinese and the French participants. Uninformative cases are those where the percentage of coherent responses is 1.0 for the French and the Chinese participants. The bars indicate the 95% confidence intervals for proportions.

Figures 1–3^3^ show the comparison of the percentage of coherence for the Chinese and the French participants in inference schemas PMP, PAC, and PIF in the sufficient conditional. The *Z*-test for comparing two proportions was used to compare the coherence for the Chinese and the French participants.

We see on the abscissa all the combinations of the probabilities of the conditional and the probabilities of the antecedent, and on the ordinate, the percentage of coherent response for the Chinese and the French participants. For example, when the probability of the conditional is 1, and the probability of the antecedent is high, the percentage of coherent response is 87% for the French and 89% for the Chinese. There is no significant difference between the percentage of coherent responses for the Chinese and the French participants.

In PMP, it is to be noted that, in 6 out of 16 cases, the inference schema is called probabilistically uninformative as all responses are considered coherent, the coherence interval being [0, 1]. Figure 1 shows that in 10 informative cases, overall, there is no significant difference in coherence between the Chinese and the French participants. The only significant difference (*p* < 0.05) concerns the case where the probability of conditional is low, and the probability of antecedent is 100%. In this case, the percentage of coherence is higher for the French participants than for the Chinese participants.

Figure 2 shows that in 13 informative cases in PAC, there are three significant differences (*p* < 0.05) in the percentage of coherent responses between the Chinese and the French participants. For PIF, Figure 3 indicates only two significant differences (*p* < 0.05) in the percentage of coherence between the Chinese and the French participants in 12 informative cases.

In total, among the 35 informative cases in the three inference schemas, we observed only six significant differences in the percentage of coherence between the Chinese participants and the French participants, three in favor of the Chinese and three in favor of the French. Given that the connector of the sufficient conditional is present and the sufficient conditional is widely used in both languages, the result is in line with the expectation: there is, overall, no significant difference between the percentage of coherence for the Chinese participants and the French participants.

##### Necessary Conditional

**FIGURE 4:**
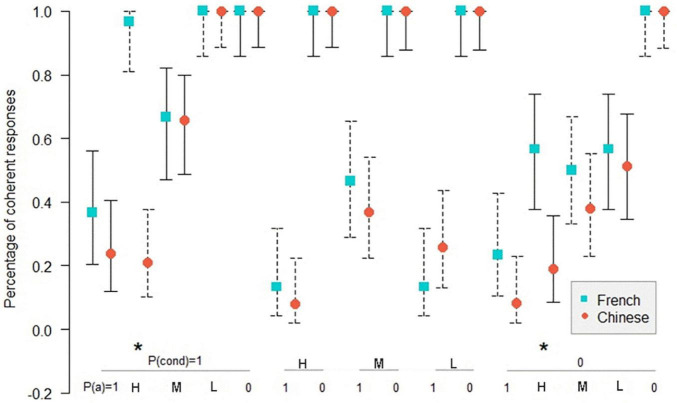
Percentage of coherent responses for the Chinese and the French participants in PMP in the necessary conditional. *: *p* < 0.05. On the abscissa, the probabilities of the conditional and the probabilities of the antecedent below; on the ordinate, the percentage of coherent response for the Chinese and the French participants. Uninformative cases are those where the percentage of coherent responses is 1.0 for the French and the Chinese participants. MP: *P*(cond) = 1, *P*(a) = 1; DA: *P*(cond) = 1, *P*(a) = 0. The bars indicate the 95% confidence intervals for proportions.

**FIGURE 5:**
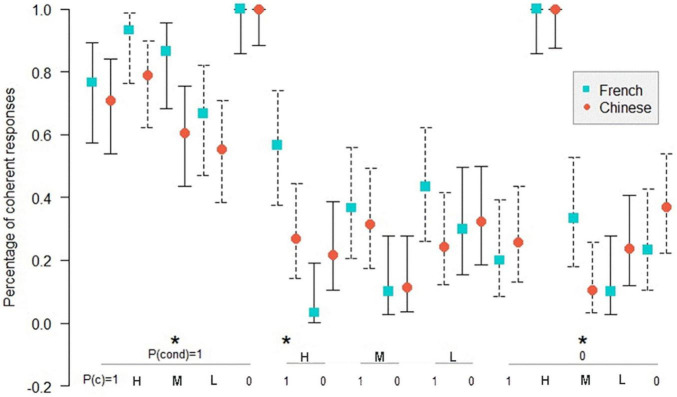
Percentage of coherent responses for the Chinese and the French participants in PAC in the necessary conditional. *: *p* < 0.05. On the abscissa, the probabilities of the conditional and the probabilities of the consequent below; on the ordinate, the percentage of coherent response for the Chinese and the French participants. Uninformative cases are those where the percentage of coherent responses is 1.0 for the French and the Chinese participants. MT: *P*(cond) = 1, *P*(c) = 0; AC: *P*(cond) = 1, *P*(c) = 1. The bars indicate the 95% confidence intervals for proportions.

**FIGURE 6:**
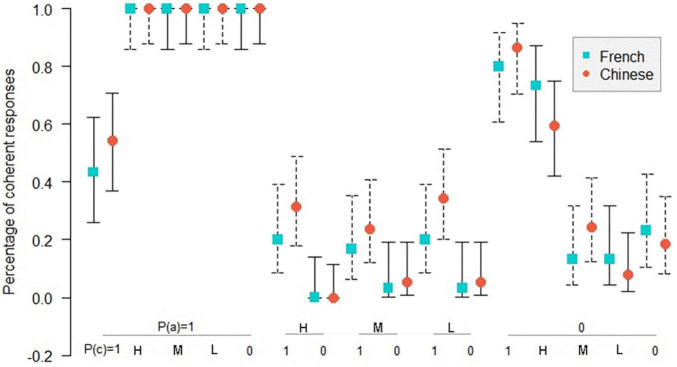
Percentage of coherent responses for the Chinese and the French participants in PIF in the necessary conditional. On the abscissa, the probabilities of the antecedent and the probabilities of the consequent below; on the ordinate, the percentage of coherent response for the Chinese and the French participants. Uninformative cases are those where the percentage of coherent responses is 1.0 for the French and the Chinese participants. The bars indicate the 95% confidence intervals for proportions.

Figures 4–6 show the comparison of the coherence percentage between the Chinese and the French participants in inference schemas PMP, PAC, and PIF in the necessary conditional. The *Z*-test was used to compare the percentage of coherence between the Chinese and the French participants.

Figure 4 shows that in 10 informative cases in PMP, there are 2 significant differences (*p* < 0.05) in coherence between the Chinese and the French participants. As indicated in Figure 5, in 14 informative cases in PAC, there are 3 significant differences (*p* < 0.05) in the percentage of coherent response between the Chinese and the French participants. Figure 6 illustrates no significant difference of coherent response for PIF between the Chinese and the French participants in 12 informative cases.

In total, among 36 informative cases in the three inference schemas, we observed only five significant differences between Chinese and the French participants, all in favor of the French. This result disproves our hypothesis that there should be better performance for the Chinese compared to the French, so the presence of the connector of the necessary conditional in the Chinese language, as opposed to the French language, did not give the Chinese participants an advantage over the French participants.

#### Comparison of Coherence Between the Sufficient Conditional and the Necessary Conditional

To know if the participants are really coherent in a given situation, we need to examine whether the coherence percentage for the Chinese and the French participants is above the success rate by chance. Before that, we should determine the success rate by chance. For example, in the presence of uncertainty, the participants are asked to evaluate three propositions *A*, *B*, and *C*. They have seven possible responses, *A*; *B*; *C*; *A* and *B*; *B* and *C*; *A* and *C*; *A, B*, and *C*. Supposing that *A*, *B* are in the coherence interval, *C* is not in the coherence interval, then, we have three possible coherent responses: *A*; *B*; *A* and *B*. The success rate by chance to give a coherent response is 3/7. Supposing now that only *A* is in the coherence interval among the seven possible responses, the success rate by chance is then 1/7. When there is a combination of certainty in the statements: 0% and/or 100%, the participants are invited to evaluate two proposals: *A* and *B*. They have three possible responses: *A*; *B*; *A* and *B*. If only *A* is in the coherence interval, the success rate by chance is 1/3. Thus, for each question, we compare the coherence percentage of the participants with the success rate by chance.

In the informative cases, the X^2^ test is used to compare the rate of coherence with the success rate by chance in each inference schema of the two conditionals. Table 3 shows the number of cases where the rate of coherence is above the chance of the total number of informative cases.

**TABLE 3:** Number of cases where the rate of coherence is above chance of the total number of informative cases.

Conditional	Inference schema	French	Chinese
Sufficient conditional	PMP	10/10	9/10
	PAC	6/13	5/13
	PIF	12/12	12/12
	Total	28/35	26/35
Necessary conditional	PMP	3/10	2/10
	PAC	8/14	4/14
	PIF	2/12	5/12
	Total	13/36	11/36

It indicates that, in PMP, the coherence rate of the Chinese participants is higher than the success rate by chance in 9 of 10 informative cases in the sufficient conditional and in only 2 of 10 informative cases in the necessary conditional. Likewise, the coherence rate for the French participants is higher than the success rate by chance in all 10 informative cases in the sufficient conditional and in only 3 of 10 informative cases in the necessary conditional. According to Fischer’s exact test, the difference in performance between the sufficient conditional and the necessary conditional is significant for both the Chinese and the French. There is better performance in the sufficient conditional than in the necessary conditional. Our results in the sufficient conditional in PMP, which includes classical DA and MP, are consistent with Evans et al. (2015), who found that the scores of coherence were significantly above chance for MP and DA.

Table 3 shows that in PAC, the coherence rate for the Chinese participants is higher than the success rate by chance in 5 of 13 informative cases in the sufficient conditional and 4 of 14 informative cases in the necessary conditional. For the French participants, it happens in 6 of 13 informative cases in the sufficient conditional and 8 of 14 informative cases in the necessary conditional. According to Fischer’s exact test, there is no significant difference in performance between the sufficient conditional and the necessary conditional, neither for the Chinese nor the French. Our results in the sufficient conditional in PAC, which includes classical MT and AC, are relatively consistent with Evans et al. (2015), who found that the scores of coherence were below chance for MT and above chance for AC in one of two experimental situations. The poor performance of the Chinese and the French participants in PAC on the sufficient conditional could be explained by directionality, which plays an important role in conditional reasoning (Oberauer and Wilhelm, 2000; Evans et al., 2005; Oberauer et al., 2005). The direction of PAC (knowing the probability of conditional “if A, then C,” and the probability of C, one should deduce the probability of A) does not correspond to the direction of the conditional. PAC is, therefore, more difficult than PMP (knowing the probability of conditional “if A, then C,” and the probability of A, one should infer the probability of C), which corresponds to the direction of the conditional.

In PIF, the coherence rate for the Chinese participants is higher than the success rate by chance in all 12 informative cases in the sufficient conditional and in only 5 of 12 informative cases in the necessary conditional. That happens for the French participants in all 12 cases in the sufficient conditional and only 2 of 12 informative cases in the necessary conditional. According to Fischer’s exact test, there is a significant difference in performance between the sufficient conditional and the necessary conditional, both for the Chinese and the French. The high coherence rate in the sufficient conditional is consistent with the results of previous studies (i.e., Cruz et al., 2015).

We noted the number of coherent and not coherent responses in each situation in the sufficient conditional and the necessary conditional. We indicated the cases where the rate of coherence is above chance (see Data Availability Statement). We found that the coherence rate is very low in some situations, even though it could be higher than the success rate by chance. In Table 4, we have identified the number of cases where the coherence rate is below 50% of the total number of cases.

**TABLE 4:** Number of cases where the coherence rate is below 50% of the total number of informative cases.

		French		Chinese	
Conditional	Inference schema	Certain conditional	Uncertain conditional	Certain conditional	Uncertain conditional
Sufficient conditional	PMP	0/7	1/3 (1)	0/7	3/3 (2)
	PAC	1/7	6/6 (3)	2/7	6/6 (1)
	Total	1/14	7/9 (4)	2/14	9/9 (3)
Necessary conditional	PMP	2/7	3/3 (1)	5/7	3/3 (1)
	PAC	4/8	5/6 (3)	4/8	6/6 (2)
	Total	6/15	8/9 (4)	9/15	9/9 (3)
2 conditionals	Total	7/29	15/18 (8)	11/29	18/18 (6)

*In the brackets ( ): the number of cases where the coherent rate is below 50% but above chance. For example, 5/6 (3): among the French participants, in PAC with the necessary conditional, when the conditional is uncertain, among 6 informative cases, the coherence rate is below 50% in 5 cases, 3 of which are above chance.*

Table 4 shows that the coherence rate below 50% is found chiefly with uncertain conditional, even though it is above chance in some cases. One explanation is that our task required making relative probability judgments, which are known to be more difficult than absolute probability judgments (Stewart et al., 2005; Guest et al., 2016). This could have impaired the coherence rate of our participants in the conditions involving uncertainty.

## General Discussion

According to the weak version of the Sapir-Whorf hypothesis, namely that language influences the way of thinking, we expected a similar performance for the Chinese and the French participants in the sufficient conditional, and better performance for the Chinese participants in the necessary conditional, since a connector for the necessary conditional exists only in Chinese. However, comparing the percentage of coherence between the Chinese and the French participants in inference schemas PMP, PAC, and PIF shows no significant difference in the sufficient conditional and the necessary conditional. This result does not confirm our hypothesis.

Thus, the presence of the necessary conditional connector in the Chinese language does not give the Chinese participants an advantage in this type of reasoning compared to the French participants. The different languages implying a difference about the presence of the necessary conditional connector, more widely different grammatically based categorization, do not affect the reasoning since a difference does not follow them in reasoning performance. To explain this result, we consider that in the French language, although the connector of the necessary conditional does not exist as such, the reasoning of the necessary conditional exists by expressions less concise and formal than a connector, which seem to be as efficient as connectors yet. Our result supports the universalist hypothesis. According to the universals of grammar, there is an isomorphism in the lexical and grammatical core of the world’s languages, even if they all differ infinitely from one another, both in their structure and in their lexicon. Cross-cultural communication would be impossible if there were not, besides considerable variations, a kind of common core based on shared or equivalent words but also on shared or equivalent grammatical structures (Wierzbicka, 1993). For Wierzbicka (1993, p. 119), “It is clear that what is necessary both for a comparative study of languages and for a study of the functioning of language as a human faculty is an authentic universal perspective, and not a perspective specific to a particular language. Although every language has its own unique structure and equally unique lexicon (a lexicon that also incorporates a unique semantic structure), some areas can be considered mutually isomorphic. It is this (partial) isomorphism in grammar and lexicon that makes the notion of “linguistic universals” a legitimate notion.” Chomsky (1994) proposes a description based on phrase structure syntax and x-bar (headword) grammar. According to the theory of principles and parameters, the deep structure thus identified is part of universal grammar. The universalist hypothesis considers that logical reasoning is performed on abstract representations, which are common, universal, and products of semantic, grammatical, and pragmatic analysis, regardless of the realization of a function in the surface structure of a particular language. In fact, we agree with Politzer (1991) that connectors in one language will operate in all languages because they stem from general principles of human communication. However, the Sapir-Whorf hypothesis could not be categorically refuted in the field of conditional reasoning. Indeed, although it has not been confirmed in most research (Brown et al., 1980; Au, 1983; Zepp, 1983; Zepp et al., 1987; Politzer, 1991; Cara and Politzer, 1993), Yeh and Gentner (2005) partly validated its weak version. More generally, some experimental studies on color perception, spatial cognition, and spatial representation of events in time support the weak version of linguistic relativity theory (for recent reviews, see Pederson, 2007; Everett, 2013). Furthermore, concerning the weak version of the Sapir-Whorf hypothesis, one of the difficulties is to isolate the effects of language from the impact of culture. Indeed, the role of culture in thinking is undeniable (Nisbett et al., 2001; Hiroshi et al., 2007; Hiroshi, 2016; Nakamura et al., 2018).

As claimed by our second hypothesis, there should be a better performance in the sufficient conditional than in the necessary conditional for both the Chinese and the French. We consider that despite isomorphism of the sufficient conditional and the necessary conditional, the two conditionals might involve different processes, resulting in differences in reasoning performance in favor of the sufficient conditional. In fact, on the logical aspect, the necessary conditional “Only if A, C” is equivalent to the sufficient conditional “If not-A, then not-C”, thus, the sufficient conditional and the necessary conditional could be considered as isomorphic. Nevertheless, our results show an important difference in favor of the sufficient conditional compared to the necessary conditional. Precisely, in the PMP and the PIF inference schemas, the Chinese and the French participants are coherent in the sufficient conditional, which is not the case of the necessary conditional. In the PAC inference schema, the number of situations where participants are coherent is quite close in the sufficient conditional and the necessary conditional. This result confirms our hypothesis in the PMP and the PIF inference schemas that predicted better performance in favor of the sufficient condition.

We first examined PMP and PAC inference schemas in the sufficient conditional and the necessary conditional. The probability of the sufficient conditional *P*(If A, then C) is *P*(C|A), but the probability of the necessary conditional *P*(Only if A, C) is not *P*(C|A). When the probability of the necessary conditional *P*(Only if A, C) is 100%, the inference is clear; participants can infer directly without going through the sufficient conditional. But when the probability of the necessary conditional *P*(Only if A, C) is not 100%, the participants very likely need to transform the necessary conditional into the sufficient conditional. Indeed, a necessary condition does not guarantee any event, and it does not lead to another result in general. If, in addition, we apply a probability to this conditional, it is very difficult to make PMP, PAC, or PIF inferences. For example, the probability of the necessary conditional “Only if A, C” is low, the probability of A is 0%, the participants must choose the probability of C: below low, just low, or above low. According to these elements, we think it is very likely that the participants would transform *a priori* the necessary conditional into a sufficient conditional before the reasoning process. The exceptional case is where the probability of the necessary conditional “Only if A, C” is 100%. In this condition, if the probability of A is 0%, one can infer that the probability of C is 0%; if the probability of C is 100%, one can deduce that the probability of A is 100%; one can also make other PMP and PAC inferences from the verbal probabilities of the second premise.

Normally, the interpretation of the necessary conditional “Only if A, C” is the sufficient conditional “If not-A, then not-C,” but it is not known if this is the actual interpretation of the participants. Indeed, the mental load to transform the necessary conditional “Only if A, C” to the sufficient conditional “If not-A, then not-C” is rather high because of the presence of negation in the sufficient conditional. The polarity effect (affirmative or negative), as the directionality effect, has been demonstrated in studies of conditional reasoning. For example, research by Grosset and Barrouillet (2003) showed that affirmative inferences took less time to endorse than denial inferences. Evans et al. (2015) found that coherent rates are better for affirmative inferences than negative inferences. The mental load is heavier with one negation; it might be even more with double negation. It is unlikely that the participants will do such a costly transformation. Instead, the most likely transformation of the necessary conditional “Only if A, C” would be the sufficient conditional “If C, then A.” In addition, some participants spontaneously told us that they made this interpretation. We analyzed the coherence of the participants with the transformation of “Only if A, C” to “If C, then A.” We noted the number of coherent and not coherent responses in each situation in the necessary conditional and indicated the cases where the rate of coherence is above chance (see Data Availability Statement). The result shows a better performance for the French participants and slightly better for the Chinese participants than the transformation of “Only if A, C” to “If not-A, then not-C.” The number of cases where the rate of coherence is above the chance of the total number of informative cases goes from 13/36 to 21/33 for the French participants and from 11/36 to 15/33 for the Chinese participants. With the transformation of “Only if A, C” to “If C, then A,” PMP in the necessary conditional *P*(Only if A, C), *P*(A) = > *P*(C) presents two additional difficulties compared to PMP in the sufficient conditional: the transformation of the necessary conditional into the sufficient conditional, and the directionality in the transformed PMP: *P*(If C, then A), *P*(A) = > *P*(C). This is what makes this inference schema particularly difficult. In PAC, in the sufficient conditional *P*(If A, then C), *P*(C) = > *P*(A), there is the difficulty of directionality in comparison with PMP in the sufficient conditional. On the other side, in PAC, in the necessary conditional *P*(Only if A, C), *P*(C) = > *P*(A) transformed into *P*(If C, then A), *P*(C) = > *P*(A), there is the difficulty of the transformation compared to PMP in the sufficient conditional. So, according to this analysis, among the 4 cases of PMP and PAC in both conditionals, the PMP in the sufficient conditional is the easiest; the PMP in the necessary conditional is the most difficult. The result provided in Table 3 confirms this. Indeed, the number of cases with coherence above chance in PMP in the sufficient conditional is very high: 10/10 for the French, and 9/10 for the Chinese; whereas the number of cases of coherence above chance in PMP in the necessary conditional is meager: 3/10 for the French, and 2/10 for the Chinese. The number of cases with coherence above chance in PAC is moderately low, in the sufficient conditional: 6/13 for the French, 5/13 for the Chinese; in the necessary conditional: 8/14 for the French, and 4/14 for the Chinese.

We then studied the PIF inference schema in the two conditionals. The number of cases with coherence above chance in PIF in the sufficient conditional is very high: 12/12 for the French and Chinese, whereas it is much lower in the necessary conditional: 2/12 for the French and 5/12 for the Chinese. On one side, the fact that PIF works in the sufficient conditional but not in the necessary conditional indicates that the different conditional connectors play an important role in this inference, therefore the predominant role of semantics, which supports the position of the inferential conditional. On the other side, the good performance of the participants in PIF in the sufficient conditional confirms the position of the probability conditional, showing the important effect of the general pragmatic. Nevertheless, one might ask why the participants can perform PIF in the sufficient conditional but not in the necessary conditional. In fact, PIF having no semantic connection, it can work in the sufficient conditional, which is simple, direct, and much closer to conjunction than the necessary conditional. Moreover, in the sufficient conditional, it is easy to obtain *P*(C|A) = *P*(C), which explains excellent performance from the participants. In contrast, the necessary conditional is more complex and very likely needs to be transformed beforehand into the sufficient conditional. As argued previously, the most likely transformation of the necessary conditional “Only if A, C” would be “If C, then A.” Thus, *P*(Only if A, C) would be transformed to *P*(if C, then A), so into *P*(A|C). In addition, there is also the question of order. With the probabilities being given in the order *P*(A) and *P*(C), it is more natural to consider the first statement as an antecedent, the second as a consequent. Then, it is easier to go to *P*(C|A) than to *P*(A|C), making PIF in the necessary conditional more difficult than in the sufficient conditional. In short, from *P*(A), *P*(C), without semantic connection between them, the participants with their experiences, intuitions, and general pragmatic can go to the probability of the sufficient conditional, but hardly go to the probability of the necessary conditional. Indeed, the path of the PIF in the sufficient conditional is *P*(A), *P*(C) = > *P*(C|A). Compared to this path, in the necessary conditional, to make the inference *P*(A), *P*(C) = > *P*(Only if A, C), two additional steps would be required: change of order between *P*(A) and *P*(C), and transformation of *P*(Only if A, C) to *P*(If C, then A), which allows reaching *P*(C), *P*(A) = > *P*(A|C). This comparison of PIF between the two conditionals helps us understand the difficulty of PIF in the necessary conditional.

Therefore, from the analysis of three inference schemas in the sufficient conditional and the necessary conditional, we can say that the two conditionals can be considered isomorphic. Still, their information processing is different: very likely, further transformation steps, the problem of directionality, and the problem of order have made inferences schemas PMP and PIF more difficult in the necessary conditional.

Finally, we addressed the limits of our work.^4^ To avoid random responses, we decided not to combine two uncertain premises, while keeping a large spectrum of the level of uncertainty in the remaining premise. It is essential to combine two uncertain premises in the design of the experiment. Indeed, it might be interesting to include this situation to study the coherent rates in all situations. In this study, we chose to represent “objective” certainty (De Finetti, 1980) by numerical values 0% and 100%. To represent uncertainty, we used verbal labels. This choice allowed us to take into consideration the first epistemic level described by De Finetti with the idea that, in the first instance, the intuition of the probability of occurrence of an event is qualitative and can be positioned on an ordinal scale but also likely to be compared with another event (Baratgin and Politzer, 2016). The second level corresponds to quantitative evaluation (De Finetti, 1964). This choice, however, can be discussed. We assumed that degrees of qualitative belief are naturally verbalizable in language by many expressions and that these expressions are a natural and appropriate format for communicating probability. We take the fact that they are imprecise as a reflection of how they can be mentally represented. However, using a mix of numerical (for certainty) and verbal (for uncertainty) scales can pose some challenges (Jenkins et al., 2018). Several studies have shown differences in the interpretation of verbal probabilities when reported in quantitative values. For example, people tend to interpret certain verbal statements in an extreme way (Teigen et al., 2013) or to interpret expressions referring to a serious event as indicating a higher probability than those referring to a more neutral event (Harris and Corner, 2011). These variations even appear to be greater with Chinese than Western participants (Harris et al., 2013). However, in this study, the participants were asked to respond without converting their probability judgment numerically. This suggests that the participants remained at the verbal level, without moving to the meta (quantitative) level. The correspondence of the quantitative values 0% and 100% with “certainly false” and “certainly true” should be quite immediate and should not lead to any problems. Nevertheless, there is another way to represent the imprecision of qualitative degrees of belief using probability intervals (as opposed to point premise probabilities). Indeed, there are extensions of coherence formulas to interval premise probabilities (for a review, see Kleiter, 2018). It would, therefore, be interesting to replicate our experiment using this probability interval format to represent the uncertainty of the premises. In addition, we decided to study the coherent rate globally in this paper. Individual differences were not investigated as the participants did not have to deal with the same questions. It would be relevant to study such differences in our future project.

In summary, the framework of the new paradigm, more precisely the Finettian approach, allowed us to take into account uncertainty in human reasoning. Also, the use of qualitative probability allowed us to be closer to reality than numerical probability in the research of conditional reasoning. We found that, in some situations, the coherence rate is very low, it is possible that relative probability judgments are more difficult to process than absolute judgment. Although we are convinced of the validity of our method, it would be interesting to propose a numerical probability in a future study for comparison. The new paradigm model is interesting but could not explain the incoherent responses of the participants that are numerous and not negligible. So, we suggest that the different pragmatic aspects in information processing should be better taken into consideration to describe and evaluate human rationality. In addition, through this study on the comparison between the sufficient conditional and the necessary conditional, we think that apart from the models of the new paradigm, other forms of logic should also be studied not to neglect the semantic aspect.

## Data Availability Statement

The datasets analyzed for this study can be found in the Open Science Framework repository at the following address: https://osf.io/zawyj/.

## Ethics Statement

The studies involving human participants were reviewed and approved by M. Maudinet Marc, Docteur en anthropologie, Ancien Directeur pédagogique du Master Gestion et Politiques du Handicap Sciences Po de Paris, Consultant indépendant expert auprès du Conseil de L’Europe, Président du Conseil Scientifique de la FISAF. M. Gutnik Fabrice, Enseignant-chercheur associé, Chercheur associé CURAPP, Université Jules Verne Amiens. Consultant en ressources humaines. Mme Aitao Tang, Ingénieur en informatique, diplômée de l’université de Paris VI, SOPRA STERIA. M. Daniel Morfouace, Professeur de philosophie, université Lille. The patients/participants provided their written informed consent to participate in this study.

## Author Contributions

JS and JB contributed to conceptual elaboration, design of the study, and draft of the manuscript. JS and DT contributed to data collection. JS contributed to data analysis. All authors contributed to the article and approved the submitted version.

## Conflict of Interest

The authors declare that the research was conducted in the absence of any commercial or financial relationships that could be construed as a potential conflict of interest.

## Publisher’s Note

All claims expressed in this article are solely those of the authors and do not necessarily represent those of their affiliated organizations, or those of the publisher, the editors and the reviewers. Any product that may be evaluated in this article, or claim that may be made by its manufacturer, is not guaranteed or endorsed by the publisher.

## Footnotes

1 Thus, the variant schema of PIF called “centering” or the “conjunctive sufficiency” schema: “A and C, therefore if A then C” will be not studied (for studies of this schema, see, for example, Cruz et al., 2015, 2016; Politzer and Baratgin, 2016; Vidal and Baratgin, 2017; Skovgaard-Olsen et al., 2019).

2 There are stereotypes about gender and first names (Coulmont and Simon, 2019). For example, a stereotype might be that housework is more women’s business. Thus, participants might make the shortcut during their reasoning: men are in the living room, while women are in the kitchen. To avoid this, we indicate that “Sophie is in the living room; Mary is in the kitchen.” We took the same precautions for the first names. Some studies show that the choice of the first name can be an indicator of social origin (Charonnat, 2017).

3 There is no difference between straight and dashed confidence intervals in the figures. The two types of lines have been used alternately to facilitate reading.

4 We thank the reviewer NC for pointing out these important points and also on the idea of using probability intervals for the premises.
